# Early Altered Resting-State Functional Connectivity Predicts the Severity of Post-Traumatic Stress Disorder Symptoms in Acutely Traumatized Subjects

**DOI:** 10.1371/journal.pone.0046833

**Published:** 2012-10-02

**Authors:** Yan Zhou, Zhen Wang, Ling-di Qin, Jie-qing Wan, Ya-wen Sun, Shan-shan Su, Wei-na Ding, Jian-rong Xu

**Affiliations:** 1 Department of Radiology, Ren Ji Hospital, Jiao Tong University School of Medicine, Shanghai, People’s Republic of China; 2 Shanghai Mental Health Center, Shanghai Jiao Tong University School of Medicine, Shanghai, People’s Republic of China; 3 Department of Neurosurgery, Ren Ji Hospital, Jiao Tong University School of Medicine, Shanghai, People’s Republic of China; University of South Florida Alzheimer’s Institute, United States of America

## Abstract

The goal of this study was to investigate the relationship between resting-state functional connectivity and the severity of post-traumatic stress disorder (PTSD) symptoms in 15 people who developed PTSD following recent trauma. Fifteen participants who experienced acute traumatic events underwent a 7.3-min resting functional magnetic resonance imaging scan within 2 days post-event. All the patients were diagnosed with PTSD within 1 to 6 months after trauma. Brain areas in which activity was correlated with that of the posterior cingulate cortex (PCC) were assessed. To assess the relationship between the severity of PTSD symptoms and PCC connectivity, contrast images representing areas positively correlated with the PCC were correlated with the subject’s Clinician-Administered PTSD Scale scores (CAPS) when they were diagnosed. Furthermore, the PCC, medial prefrontal cortex and bilateral amygdala were selected to assess the correlation of the strength of functional connectivity with the CAPS. Resting state connectivity with the PCC was negatively correlated with CAPS scores in the left superior temporal gyrus and right hippocampus/amygdala. Furthermore, the strength of connectivity between the PCC and bilateral amygdala, and even between the bilateral amygdala could predict the severity of PTSD symptoms later. These results suggest that early altered resting-state functional connectivity of the PCC with the left superior temporal gyrus, right hippocampus and amygdala could predict the severity of the disease and may be a major risk factor that predisposes patients to develop PTSD.

## Introduction

Post-traumatic stress disorder (PTSD) is an anxiety disorder that can develop following exposure to a traumatic event, such as military combat, traffic accidents, rape, assault, or natural disasters. It is a complex syndrome that involves re-experiencing of symptoms (e.g., nightmares and flash-backs), hyperarousal symptoms (e.g., insomnia), numbing symptoms (e.g., restricted affect and anhedonia), and avoidance symptoms (e.g., avoiding trauma-related stimuli), in addition to cognitive impairment, such as poor concentration and difficulty in explicitly recalling aspects of the traumatic event [1]. More than one third of people with PTSD fail to recover, even after many years [2]. Additionally, 50% of PTSD patients have comorbid drug abuse and other mental disorders, and their suicide rate is six times that of normal individuals [3]. Therefore, how to reduce the damage to human health and the large consumption of social rescources caused by PTSD is becoming a scientific cutting-edge issue primarily focused by the government and scientific community. A deeper understanding of the neurobiological basis of PTSD may also explain individual differences in susceptibility to the disorder and aid in the development of more effective treatments.

Over the past decade, neuroimaging techniques have been critical in the process of identifying important brain systems in the pathophysiology of PTSD. Specifically, findings from functional neuroimaging studies indicated abnormalities in amygdale and amygdala-linked circuitry involving the medial prefrontal cortex (mPFC), insula, anterior cingulate cortex (ACC), and hippocampus [4], [5], [6], [7], [8] Studies [5], [6], [7], [8] have shown heightened amygdala responsivity in PTSD during symptomatic states and during the processing of trauma-unrelated affective information. Importantly, amygdala responsivity is positively associated with the severity of symptoms in PTSD [9], [10], [11], [12], [13]. In contrast, the mPFC responsivity is negatively associated with the severity of PTSD symptoms [9], [14], and the mPFC appears to be hyporesponsive during symptomatic states and the performance of emotional cognitive tasks in PTSD.

Resting-state functional connectivity has been widely used in the study of PTSD [9], [10], [11], [12], [13], [14], [15], [16], because during scanning, the absence of demanding cognitive activity and instructions makes it more straightforward to compare brain activity across groups that may differ in motivation or cognitive abilities. It is unknown whether the structural and functional changes are due to the effect of the traumatic event on neural function, or rather represent underlying risk factors that predate the trauma and predispose individuals to developing PTSD. To partially assess this topic, we aimed to assess the relationship between resting-state functional connectivity and clinical severity of PTSD in patients who developed PTSD following recent trauma.

## Materials and Methods

### Subjects

The current study included 15 car accident victims randomly recruited from the Emergency Department of Renji Hospital. Most of them witnessed actual or threatened death or serious injury to others, and some of them had mild concussive neurotrauma and bruises. In order to guarantee scanning quality, to avoid major head movements during data acquisition, and to eliminate the potential effect of lesions in the brain on the analysis of resting state functional connectivity, we excluded the patients with significant head injury. All subjects underwent baseline evaluation within 2 days (2d). The tests included the Mini-International Neuropsychiatric Interview (MINI) [17], Acute Stress Disorder Structured Interview (ASDI) [18] and functional magnetic resonance imaging (fMRI) scans. Follow-up evaluation for the PTSD diagnosis, based on the Clinician-Administered PTSD Scale (CAPS) [19], was conducted at 1 and 6 months post-accident. All PTSD subjects fulfilled criterion for PTSD as assessed using CAPS, either 1 month or 6 months post-accident. All participants were right-handed.

The exclusion criteria were as follows: (1) younger than 18 or older than 60 years, with an education <9 years; (2) ASDI <3; (3) significant head injury (i.e., abnormalities on conventional MRI, neurological abnormalities during emergency department evaluation, and loss of consciousness longer than several seconds during the accident); (4) a history of neurological disorders; (5) current axis I disorders at the time of the accident, as assessed using the MINI [17], or drug or alcohol abuse/dependence within 6 months prior to the accident; (6) medications (psychotropic drugs within 4 weeks prior to scanning); and (7) contraindications to MRI.

The current study was approved by the Research Ethics Committee of Renji Hospital. All subjects gave their informed written consent. All procedures were in accordance with institutional guidelines.

### MRI Acquisition

MRI was performed on a 3T magnetic resonance scanner (GE Signa HDxt 3T, USA). A standard head coil with foam padding was used to restrict head motion. During resting-state fMRI, the subjects were instructed to keep their eyes closed, remain motionless, and not to think of anything in particular. A gradient-echo echo-planar sequence was used to acquire functional images (repetition time [TR] = 2000 ms, echo time [TE] = 30 ms, field of view [FOV] = 230 mm^2^×230 mm^2^, matrix = 64×64, thickness = 4 mm, and gap = 0). Each fMRI scan lasted 440 s. Other sequences were also acquired, including: (1) sagittal T1-weighted 3D-magnetization prepared rapid acquisition gradient echo sequences (TR = 9.4 ms, TE = 4.6 ms, flip angle = 15°, slice thickness = 1 mm, gap = 0 mm, FOV = 256 mm×256 mm, matrix = 256×256, and slices = 155); (2) axial T1-weighted fast field echo sequences (TR = 331 ms, TE = 4.6 ms, FOV = 256 mm ×256 mm, slice thickness = 4 mm, gap = 0, slices = 34, and matrix = 512×512); and (3) axial T2-weighted turbo spin-echo sequences (TR = 3013 ms, TE = 80 ms, FOV = 256 mm×256 mm, slice thickness = 4 mm, gap = 0, slices = 34, and matrix = 512×512).

### Image Analysis

Brain MR imagings (T1-weighted and T2-weighted images) were evaluated by two experienced neuroradiologists. No gross abnormalities were observed in the participants. Functional MRI preprocessing was carried out using Data Processing Assistant for Resting-State fMRI (DPARSF V 2.0, by YAN Chao-Gan, http://www.restfmri.net), which is based on MRIcroN (by Chris Rorden, http://www.mricro.com), statistical parametric mapping (SPM5; Wellcome Department of Imaging Neuroscience, London, UK), and the Resting-State fMRI Data Analysis Toolkit (REST V1.5 software, by SONG Xiao-Wei et al., http://www.restfmri.net). The first 10 volumes of each functional time series were discarded because of instability of the initial MRI signal and the initial adaptation of participants to the situation. Data from each fMRI scan contained 220 time points, and the remaining 210 images were preprocessed. The images were subsequently corrected for slice timing and realigned to the first image for rigid-body head movement correction. No participant had motion of more than 1 mm with maximum translation in *x*, *y*, or *z*, or 1° of any angular motion throughout the course of scan. The functional images were normalized into standard stereotaxic anatomical Montreal Neurological Institute space. The normalized volumes were resampled to a voxel size of 3 mm×3 mm×3 mm. The echo-planar images were spatially smoothed using an isotropic Gaussian filter of 4 mm full width at half maximum.

Each voxel’s time-series was detrended to correct for lineral drift over time. Nine nuisance covariates (time-series predictors for global signal, white matter, cerebrospinal fluid, and the six movement parameters, including the first derivative, obtained during realignment to account for motion-related effects in blood oxygenated level-dependent) were sequentially regressed from the time-series [20]. Following this procedure, temporal filtering (0.01 Hz–0.08 Hz) was applied to the time series of each voxel to reduce the effect of low-frequency drifts and high-frequency noise [21], [22], [23].

The PCC template, which consisted of Brodmann’s areas 29, 30, 23, and 31, was selected as the region of interest (ROI) using WFU-Pick Atlas software [24]. The BOLD time series of the voxels within the seed region were averaged to generate the reference time series.

For each subject and seed region, a correlation map was produced by computing the correlation coefficients between the reference time series and the time series from all the other brain voxels. Correlation coefficients were then converted to *z* values using Fisher’s *z*-transform to improve the normality [22]. The individual *z* value was entered into a random effect one-sample t-test in a voxel-wise manner to determine brain regions showing significant connectivity to each seed region within PTSD patients under a combined threshold of P<0.01 and cluster size n = 486 mm^3^. This yielded a corrected threshold of P<0.05, determined by Monte Carlo simulation with the program AlphaSim in AFNI with the following parameters: full width at half maximum = 4 mm, within the BrainMask in REST. This procedure produced significant functional connectivity z-statistic maps for the PTSD group.

To examine whether the strength of functional connectivity in the PCC varies with the severity of disease in PTSD patients, Pearson’s correlative analysis was performed to examine relationships between the z-values and CAPS in PTSD patients at the time that patients were diagnosed using a threshold of p<0.05 as corrected by AlphaSim. Left and right amygdala templates were selected as ROIs using WFU-Pick Atlas software [24], acting as separate seed regions. The mPFC was selected as the seed region centered at Montreal Neurological Institute coordinates of −2, 48, and −4 in a 10-mm sphere, as described in a previous study [15]. For each seed region, the BOLD time series of the voxels within the seed region was averaged to generate the reference time series. Using ROI-wise functional connectivity analysis, the correlation coefficients of the functional connectivity in each pair of seed regions were calculated and then converted to z values within correlation coefficients. Pearson’s correlative analysis was performed to examine relationships between the z-values and CAPS in PTSD patients at the time of diagnosis using a threshold of p<0.05 as corrected by AlphaSim.

## Results

### Subject Characteristics

The mean age of PTSD patients (4 females, 11 males) was 41.52±12.56 years, and the mean duration of education was 12.02±2.56 years. All subjects underwent baseline evaluation within 2 d post-accident. No patient met diagnostic criteria for current axis I disorders as assessed using the MINI, and the mean ASDI was 15.42±6.01. Follow-up evaluation for PTSD diagnosis was conducted at 1 month and 6 months post-accident. Eleven and 4 patients were diagnosed 1 month and 6 months post-accident, respectively, and the mean time from accident to PTSD diagnosis was 2.33±2.28 months. The mean CAPS when the patients were diagnosed was 44.53±15.76.

### Correlation between PCC Connectivity and CAPS

**Figure 1:**
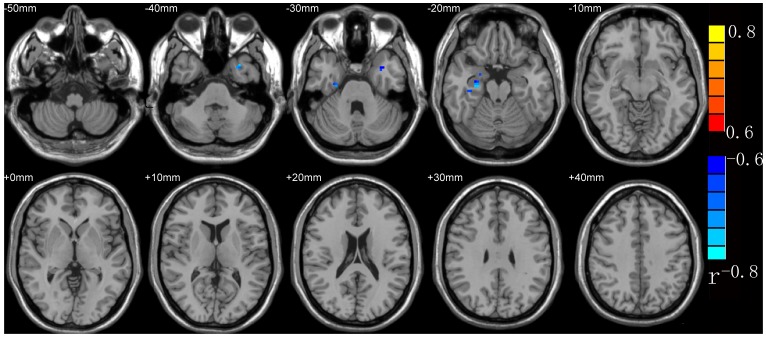
Brain regions where functional connectivity with the PCC was correlated with CAPS at the time when patients were diagnosed with PTSD. Note: The right part of the figure represents the patient’s left side. PTSD = post-traumatic stress disorder.

Connectivity with the PCC was negatively correlated with CAPS scores in the left superior temporal gyrus and right hippocampal gyrus/right amygdala (see Table 1 and Fig. 1).

**Table 1:** Brain regions where functional connectivity with the PCC correlated with CAPS scores at the time PTSD patients were diagnosed.

	Peak MNI coordinate region	Peak MNI coordinates			Number of cluster voxels
		x	y	z	
1	left superior temporal gyrus	−42	3	−24	44
2	Right hippocampal gyrus/right amygdala	36	−24	−24	61
	(p<0.05, AlphaSim-corrected)				

Note: PTSD = post-traumatic stress disorder; PCC = posterior cingulated cortex;

CAPS = the Clinician-Administered PTSD Scale.

### Correlation of Functional Connectivity within Seed Regions and CAPS

**Figure 2:**
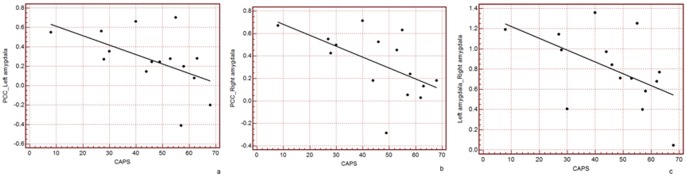
Correlation between PCC-left amygdala, PCC-right amygdala, left-right amygdala connectivity and CAPS in 15 PTSD patients: (Fig. 2a) PCC-left amygdala connectivity and CAPS, r = −0.53, p = 0.04; (Fig. 2b) PCC-right amygdala connectivity and CAPS, r = −0.57, p = 0.03; (Fig. 2c) left-right amygdala connectivity and CAPS, r = −0.54, p = 0.04.

Four regions were selected, including the PCC, mPFC and bilateral amygdala. Correlation analysis of the strength of functional connectivity within each pair of seed regions and CAPS was performed. The strengths of functional connectivity of the PCC-right amygdala (r = −0.57, p = 0.03), PCC-left amygdala (r = −0.53, p = 0.04) and right amygdala-left amygdala (r = −0.54, p = 0.04) were negatively correlated with CAPS scores in the PTSD patients at the time of diagnosis. (See Table 2 and Fig. 2).

**Table 2:** Pearson’s correlations between the PCC, mPFC, left amygdala, right amygdala and CAPS in PTSD patients.

	PCC-mPFC	PCC-L-amygdala	PCC-R-amygdala	mPFC-L-amygdala	mPFC-R-amygdala	L-amygdala-R-amygdala
r	−0.03	−0.53	−0.57	0.28	0.26	−0.54
p	0.90	**0.04**	**0.03**	0.30	0.36	**0.04**

Note: PCC = posterior cingulate cortex; mPFC = medial prefrontal cortex; CAPS = the Clinician-administered PTSD Scale; PTSD = Post-traumatic Stress Disorder; R-amygdala = right amygdala; L-amygdala = left amygdala.

## Discussion

To the best of our knowledge, this is the first study to examine the relationships between default network connectivity and prospective PTSD symptoms soon after trauma. This study demonstrated that resting state connectivity with the PCC was negatively correlated with CAPS scores in the left superior temporal gyrus and right hippocampus/amygdala. Furthermore, the strength of connectivity between the PCC and bilateral amygdala, and even between the bilateral amygdala could predict the severity of PTSD symptoms later.

These results are partly consistent with previous studies [9], [10], [11], [12], [15], [16], [25]. Structural abnormalities in the superior temporal gyrus have been found in maltreated children and adolescents with PTSD [26]. The superior temporal gyrus has connections with temporolimbic areas, including the hippocampus, amygdala, entorhinal cortex, thalamus, and neocortical association areas in prefrontal and parietal cortices [27]. The superior temporal gyrus is involved in auditory processing, including language, and has also been implicated as a critical structure in social cognition. De Bellis et al. [26] found that superior temporal gyrus volumes were larger in the chronic PTSD patients than in control subjects. They suggested that there might be a compensatory synaptic increase in the superior temporal gyrus related to the increase of the sensitivity to conditioned auditory stimuli during development, or the larger superior temporal gyrus gray matter in the PTSD subjects could be a consequence of a decreased developmentally-related input from the frontal cortex. The hippocampus is essential for the formation of stable declarative memory in humans and spatial memory in rodents, and is the brain functional domain most closely associated with learning, memory, and cognitive function [28]. Dickie et al [9] found that change in the activity of the hippocampus and subgenual anterior cingulate cortex (as a function of emotional memory) was correlated with improvement in PTSD symptoms, suggesting that activity in these areas may be associated with recovery. We found a negative correlation of the connectivity with the PCC in superior temporal gyrus and hippocampus with CAPS, suggesting that early altered resting-state functional connectivity in these areas could predict the severity of the disease and may be a major risk factor that predisposes patients to develop PTSD.

Amygdala activity plays a causal role in the experience of negative effects, such as fear, anxiety, and distress. In healthy brains, amygdala activity is thought to be dampened via top-down inhibition by the mPFC, yielding a reduction in subjective distress. However, in PTSD, a defect in mPFC function impairs inhibition of the amygdala, resulting in abnormal amygdala activity and pathological distress [5], [29]. Therefore, PTSD neuroimaging data support a model of PTSD pathogenesis that proposes two important elements: 1) the emotional distress characterizing PTSD arises from hyperactivity in the amygdala; and 2) amygdala hyperactivity is caused by defective inhibition from a hypoactive mPFC [29], [30]. A previous study [31] suggested that amygdala damage abolishes the development of PTSD among combat veterans, which supports the assertion that amygdala hyperactivity plays a causal role in the pathophysiology of PTSD. Numerous studies, using a variety of tasks and stimuli, have reported significant positive correlations between the severity of PTSD symptoms and amygdala activity [12], [13] and both positive [25] and negative [32], [33], [34] correlations with activity of the mPFC. We found that the strength of connectivity between the PCC and bilateral amygdala was negative correlated with CAPS, which may be caused by hyper-inhibition by the mPFC in the early stage of post-trauma. Early abnormal function of amygdala-linked circuitry may lead to the subsequent onset of illness. Interestingly, we also found a negative correlation of the strength of functional connectivity in right-left amygdala with CAPS. Further research is necessary to explain this phenomenon in detail.

We did not observe any significant differences in PCC connectivity to the mPFC or anterior cingulate cortex in PTSD patients at rest. This is a notable negative finding and requires replication; however, we acknowledge that it could have resulted from the following: (1) a small sample size may have led to false negative results and/or more subtle connectivity abnormalities; and (2) the resting-state task may be insensitive to detecting early and mild PCC-prefrontal and amygdala-mPFC connectivity abnormalities, which may require engagement by an overt task. Koenigs et al. [31] considered that the finding of mPFC hypoactivity in functional imaging studies of PTSD does not necessarily reflect a causal contribution to the disorder. It is possible that the mPFC hypoactivity observed in PTSD develops as a consequence of chronic distress associated with PTSD. Koenigs et al. [31] found that mPFC lesions resulted in decreased susceptibility to PTSD and they proposed that the causal role of mPFC in PTSD may be related to its function in self-insight and self-reflection. Therefore, a loss of self-insight or self-reflection may diminish the core symptoms of the disorder.

The current study has several limitations. First, the sample size was relatively small. Second, the seed-point method as the mode of analysis may have been biased by the particular seed region chosen, focusing on long-distance patterns of connectivity. However, we applied all of the ROIs mentioned in previous studies. Third, we did not obtain the patient’s fMRI again when they were diagnosed. Fourth, these connectivity differences could be resolved by other factors unrelated to the traumatic event. Fifth, we excluded the patients with significant head injury (i.e., abnormalities on conventional MRI, neurological abnormalities during emergency department evaluation, and loss of consciousness longer than several seconds during the accident). PTSD can commonly result from concussive injury resulting in loss of consciousness; thus, this criterion for exclusion severely limits generalizability. Follow-up studies should be conducted in the future to verify the present findings.

### Conclusions

This paper describes a preliminary study investigating the relationship between resting-state functional connectivity and the severity of post-traumatic stress disorder (PTSD) symptoms in people who developed PTSD following recent trauma. Early altered functional connectivity in the PCC with the left superior temporal gyrus, right hippocampus and amygdala could predict the severity of the disease, and may be a major risk factor that predisposes patients to develop PTSD.
